# Supplementary material for: OsαCA1 Affects Photosynthesis, Yield Potential, and Water Use Efficiency in Rice
Source: Int J Mol Sci. 2023 Mar 14;24(6):5560. doi: 10.3390/ijms24065560 (PMC10056782; doi:10.3390/ijms24065560)
Supplement: Supplementary file 1 [file ijms-24-05560-s001.zip › ijms-2277891-supplementary.pdf]

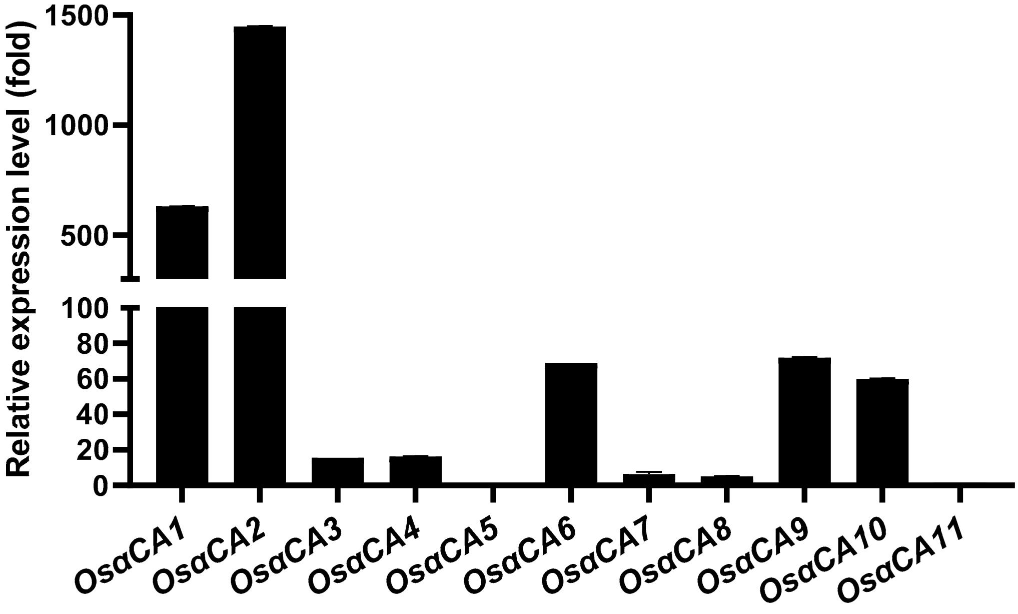

Figure S1. The relative expression levels of *OsαCAs* of rice in flag leaves. The total RNA of flag leaves was extracted to analyze the expression of alpha carbonic anhydrases of rice by qRT-PCR, consisting of *OsαCA1*, *OsαCA2*, *OsαCA3*, *OsαCA4*, *OsαCA5*, *OsαCA6*, *OsαCA7*, *OsαCA8*, *OsαCA9*, *OsαCA10* and *OsαCA11*, with *Ubiquitin* as control. Values are shown as means ± SEM, where $n = 3$.

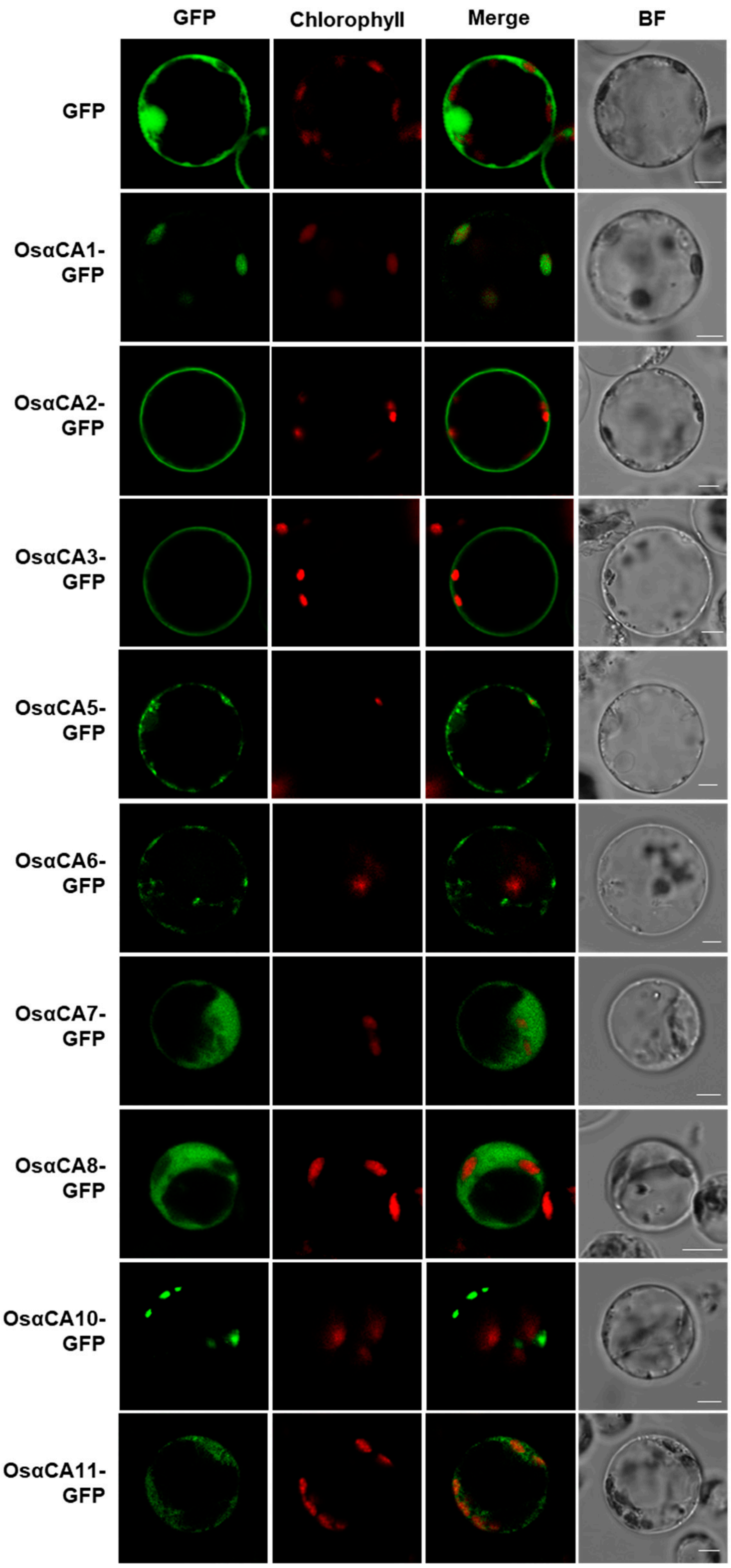

Figure S2. The subcellular localization of OsαCAs in rice. GFP—the view via GFP fluorescence; BF—the view via bright-field microscopy; chlorophyll—the view of chloroplast autofluorescence; and Merge—the merged view of GFP and chlorophyll. Scale bars: 5 μm. Scale bars: 5 μm.

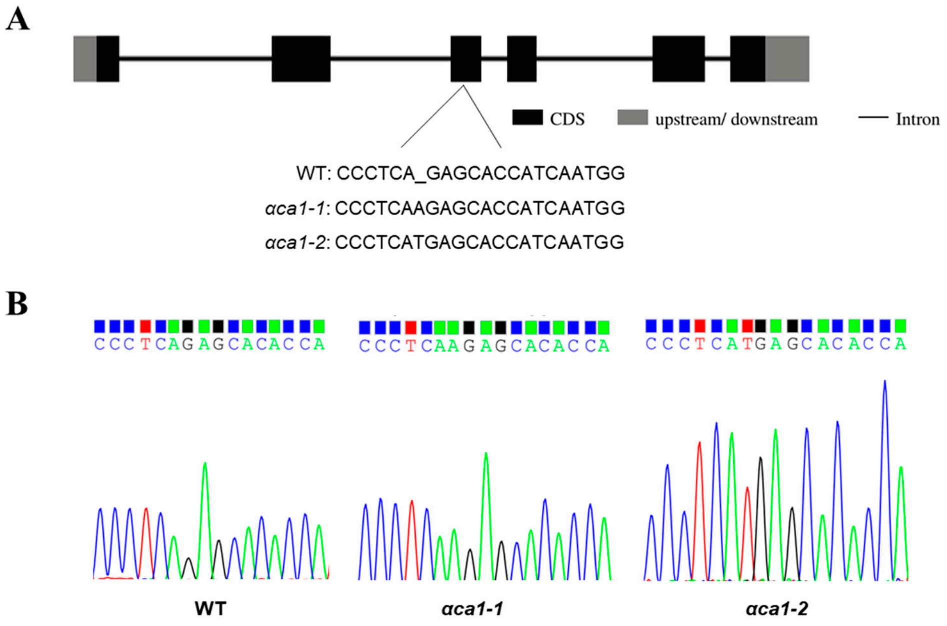

Figure S3. The gene structure and gene editing of *OsαCA1*. (A) The gene structure of *OsαCA1* and CRISPR/Cas9 editing method in *αca1* mutants. Input the CDS sequence and genomic sequence of *OsαCA1* in GSDS (*http://gsds.cbi.pku.edu.cn/*), and get the gene structure. The black rectangle represents the exons; the gray rectangle represents upstream or downstream; and the black line represents introns. B. The DNA sequencing of *OsαCA1* genome in *αca1* mutants.

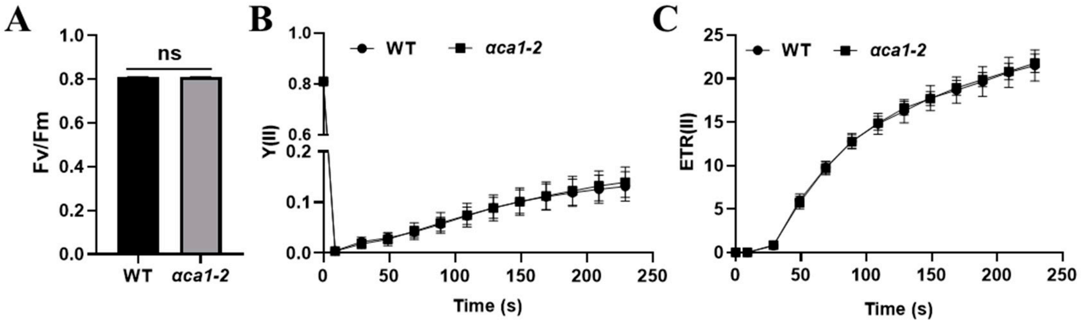

Figure S4. The activity of PSII in *αca1-2* was not affected. (A) The maximum photochemical quantum efficiency of PSII in WT and *αca1-2*. Values are shown as means ± SEM determined via Student's *t*-test, where ns indicates no significant difference, and $n \geq 10$. (B–C) The slow chlorophyll fluorescence induction kinetics of WT and *αca1-2*. (B) Actual photochemical quantum efficiency; (C) electron transport rate. The first fully expanded leaves of 2-week-old seedlings were measured. All values are shown as means ± SEM determined via Student's *t*-test, and $n \geq 18$. There were no significant differences between WT and *αca1-2*.

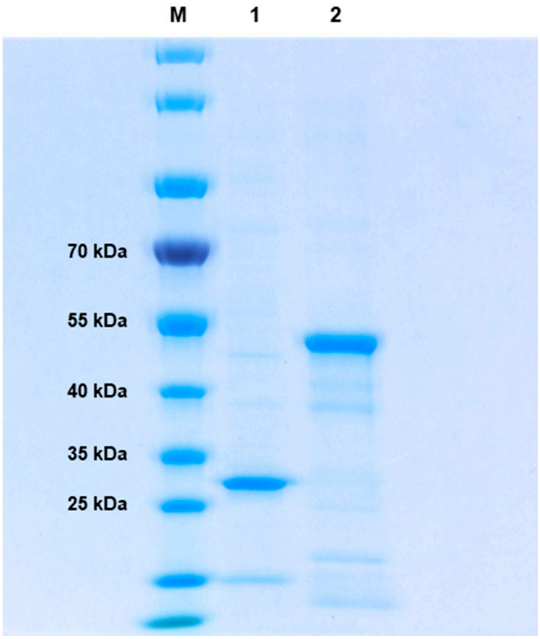

Figure S5. The purified protein with SDS-PAGE showed a bright target band. M represents the protein marker with the molecular weight at left. 1: GST protein; 2: GST-Os*α*CA1 fusion protein.

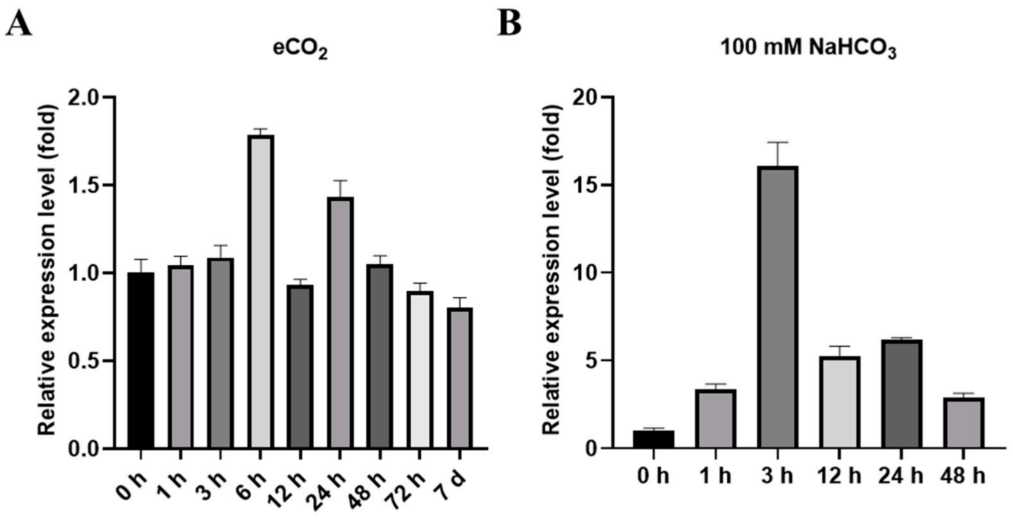

Figure S6. The expression of *OsαCA1* gene was induced by elevated CO_2 and HCO_3^- treatment. (A) The relative expression of *OsαCA1* with elevated CO_2 treatment (eCO_2). 10-day-old seedlings were treated with 1000 ppm CO_2 to detect the expression level of *OsαCA1* after 0 h, 1 h, 3 h, 6 h, 12 h, 24 h, 48 h, 72 h and 7 d. *Ubiquitin* was used as the control. Values are shown as means ± SEM, and *n* = 3. (B) The relative expression of *OsαCA1* with 100 mM $NaHCO_3$ treatment. 10-day-old seedlings were treated with 100 mM $NaHCO_3$ to detect the expression level of *OsαCA1* after 0 h, 1 h, 3 h, 12 h, 24 h and 48 h. *Ubiquitin* was the control. Values are shown as means ± SEM, and *n* = 3.

A

B

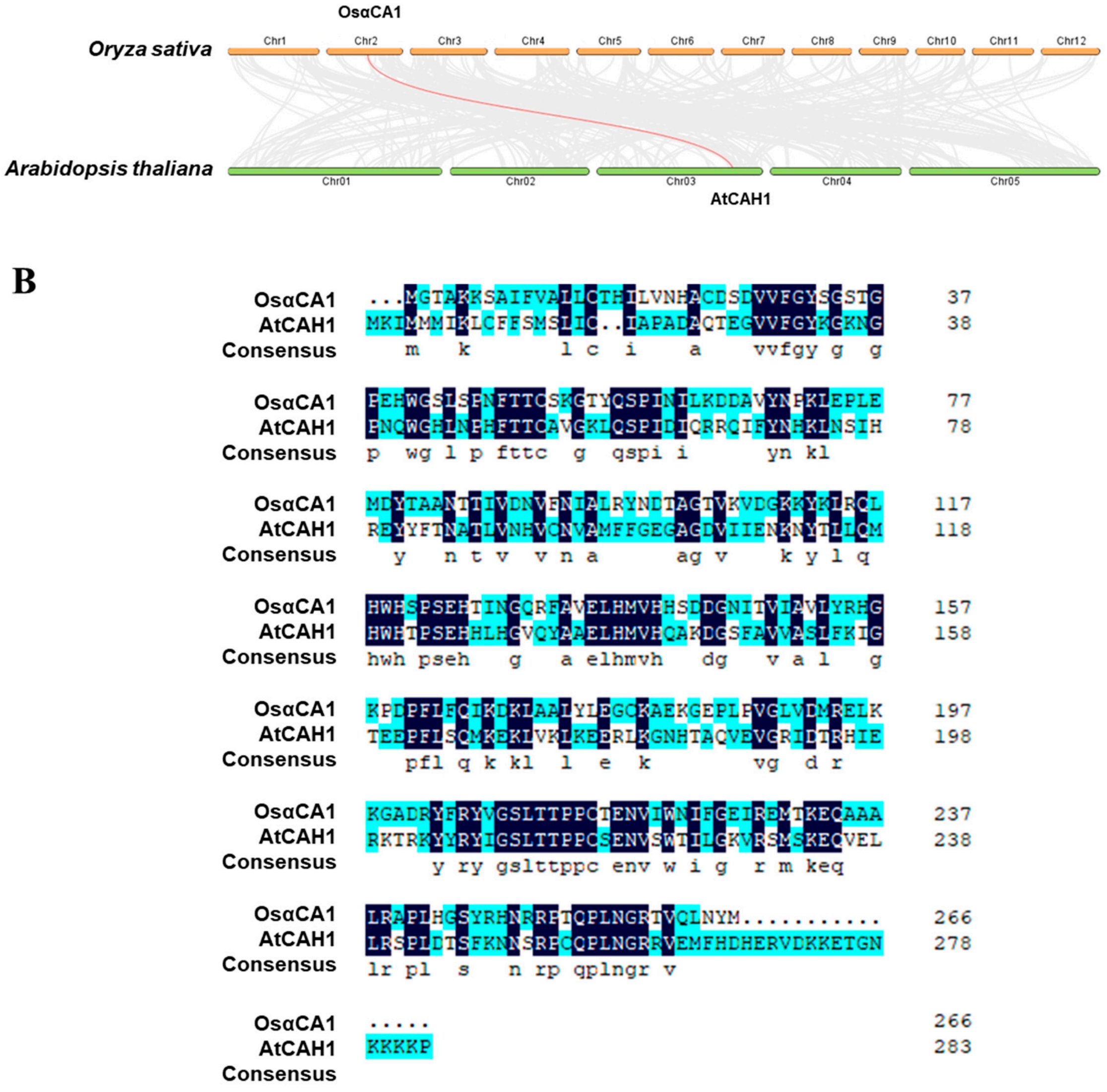

Figure S7. Os*α*CA1 was the homolog of AtCAH1 in rice. (A) The collinearity analysis of rice and Arabidopsis. Orange round-corner rectangles represent 12 chromosomes in rice; green round-corner rectangles represent 5 chromosomes in Arabidopsis; and the linked red line represents the collinearity of the two proteins. (B) The protein sequence alignment of Os*α*CA1 and AtCAH1. The protein sequences were aligned by DNAMAN, and different background colors represented different similarity levels.

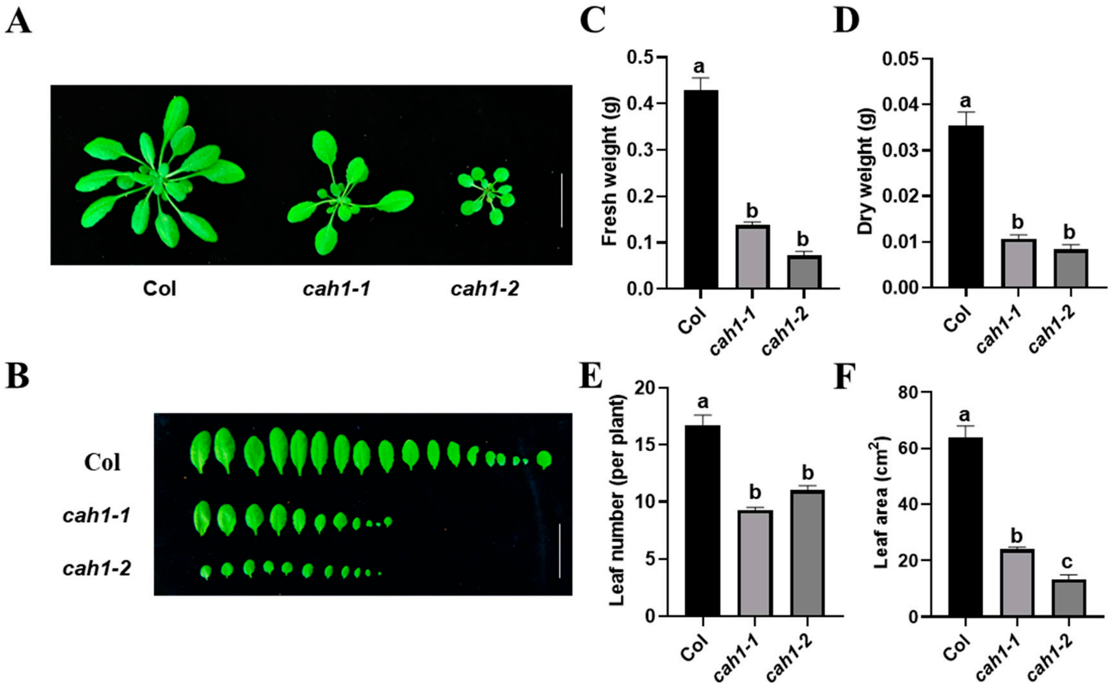

Figure S8. The knockout of *AtCAH1* inhibited plant growth. (A) The representative image of plant growth of Columbia (Col) and *cah1* mutants. Scale bar, 3 cm. (B) The representative image of rosette leaves of Col and *cah1* mutants. Scale bar, 3 cm. (C) Fresh weight of Col and *cah1* mutants. (D) Dry weight of Col and *cah1* mutants. (E) Leaf number per plant of Col and *cah1* mutants. (F) Leaf area of Col and *cah1* mutants. Values are shown as means ± SEM determined via one-way ANOVA, where different letters indicate a significant difference, $p < 0.05$, $n = 9$.

Table S1. Primers for qRT-PCR used in this study.

Gene ID	Primer name	Sequence (5' to 3')
LOC_Os02g33030	A1-RT-F	CGACACCTCCATGCACCGAA
	A1-RT-R	GGTGCGACCATTGAGTGGCT
LOC_Os04g33660	A2-RT-F	TGCTCGAACGGAAAGCAGCA
	A2-RT-R	GCAGCGCGACGTTGAAGATG
LOC_Os08g32750	A3-RT-F	GCAGATGCACTGGCACACG
	A3-RT-R	CCTGGTTCTGGGCGTTGAGG
LOC_Os08g32780	A4-RT-F	CAGCTCCGGCAAATGCACTG
	A4-RT-R	GGGTGGTGCCGATGGTGTAG
LOC_Os08g32840	A5-RT-F	GGCGGGCTGGTGATTAACGG
	A5-RT-R	GCGGTCTGGTTCTGGGTGTT
LOC_Os08g36630	A6-RT-F	TCATCAACGGCACCGCCTAC
	A6-RT-R	CCTTCTTCTCGGCGCTCTCG
LOC_Os08g36680	A7-RT-F	CCGAGATGGGCCAAGTGCAA
	A7-RT-R	TAGCCGAGGTTGCGCATCAG
LOC_Os09g28130	A8-RT-F	ACAACACCGCCTTGCACTGA
	A8-RT-R	GGCCGTGCATTTGGTTCCAC
LOC_Os09g28150	A9-RT-F	CACAAGGAGTGGGCCGTCTG
	A9-RT-R	CCGGCCTTGTAGGCTTGCAT
LOC_Os11g05520	A10-RT-F	GAGCAGGTCGCCCTCATCAC
	A10-RT-R	TGCTGTTGGGCGGGTTGTAG
LOC_Os12g05730	A11-RT-F	ACACGCTGGACCGCAACTAC
	A11-RT-R	CCAGTGGATCGCCTGGAACC
LOC_Os09g27930	UBQ-RT-F	ACCCTGGCTGACTACAACATC
	UBQ-RT-R	AGTTGACAGCCCTAGGGTG
LOC_Os02g47020	Prkase-RT-F	GAACAGGTCTCTTCCAAACT
	Prkase-RT-R	TTAAACTTTTGCCGCTTCAG
LOC_Os04g16680	SBPase-RT-F	TCTCATGTCTGCAAGTATGC
	SBPase-RT-R	TCGAATGCTACACTGAAACC
LOC_Os01g64660	FBPase-RT-F	CTCTTGAGGACGTGTTACAG
	FBPase-RT-R	GATCAAGTGTGAAGCCATTG

Table S2. Primers used for vector construction in this study.

Gene ID	Primer name	Sequence (5' to 3')	Purpose
LOC_Os04g33660	A2-GFP-F	GACTCTAGAGGATCCATGGCAGCTTCTCATGGAAAT	Subcellular localization analysis
	A2-GFP-R	GCTCACCATGGATCCCGGGGAGCTCCTCCTCTT	
LOC_Os08g32750	A3-GFP-F	GACTCTAGAGGATCCATGAGTACTTCAGCTCGCCG	
	A3-GFP-R	GCTCACCATGGATCCGTTGTTGATGAGAGGGAGAGGA	
LOC_Os08g32840	A5-GFP-F	GACTCTAGAGGATCCATGCATCGCGCACGCC	
	A5-GFP-R	GCTCACCATGGATCCTACGTTGATCAGAGGGAGAGGAAT	
LOC_Os08g36630	A6-GFP-F	GACTCTAGAGGATCCATGGGTTCGACTCGCCTC	
	A6-GFP-R	GCTCACCATGGATCCATAGCGTTTATGAGGGTAAGGC	
LOC_Os08g36680	A7-GFP-F	GACTCTAGAGGATCCATGCATTCGAGTACTCGAGC	
	A7-GFP-R	GCTCACCATGGATCCGTCCTTCTTAGGGTCTGGAATG	
LOC_Os09g28130	A8-GFP-F	GACTCTAGAGGATCCATGGCGCCACATTTCAAGAA	
	A8-GFP-R	GCTCACCATGGATCCAACTTCAAAGTACCTCACAATTC	
LOC_Os11g05520	A10-GFP-F	GACTCTAGAGGATCCATGGCGCAGATGGAGTTC	
	A10-GFP-R	GCTCACCATGGATCCGACCTTGAAGGAGATGGTGC	
LOC_Os12g05730	A11-GFP-F	GACTCTAGAGGATCCATGGTGTCTCTCCGCGC	
	A11-GFP-R	GCTCACCATGGATCCCTTGGCGAATTCCTGGAAG	
LOC_Os02g33030	A1-GFP-F	GACTCTAGAGGATCCATGGGGACTGCTAAGAAAAGTG	
	A1-GFP-R	GCTCACCATGGATCCCATGTAATTGAGCTGCACGGT	
	SP-GFP-R	GCTCACCATGGATCCTGCATGGTTCACAAGAATGT	
	CA-GFP-F	GACTCTAGAGGATCCATGTGTGACAGTGATGTGGTA	
	GST-A1-F	GTTCCGCGTGGATCCATGGGGACTGCTAAGAAAAGTG	Protein purification
	GST-A1-R	TCGACCCGGGAATTCTTACATGTAATTGAGCTGCACG	
